# Parental Preferences for Mental Health Screening of Youths From a Multinational Survey

**DOI:** 10.1001/jamanetworkopen.2023.18892

**Published:** 2023-06-20

**Authors:** Mirelle Kass, Lindsay Alexander, Kathleen Moskowitz, Najé James, Giovanni Abrahão Salum, Bennett Leventhal, Kathleen Merikangas, Michael Peter Milham

**Affiliations:** 1 Center for the Developing Brain, Child Mind Institute, New York, New York; 2 Graduate Program in Psychiatry and Behavioral Sciences, Universidade Federal do Rio Grande do Sul, Porto Alegre, Brazil; 3 Section on Negative Affect and Social Processes, Hospital de Clínicas de Porto Alegre, Universidade do Rio Grande do Sul, Porto Alegre, Brazil; 4 National Institute of Developmental Psychiatry for Children and Adolescents, São Paulo, Brazil; 5 Department of Psychiatry and Legal Medicine, Universidade Federal do Rio Grande do Sul, Porto Alegre, Brazil; 6 Lifespan Informatics and Neuroimaging Center, Philadelphia, Pennsylvania; 7 The University of Chicago, Chicago, Illinois; 8 Genetic Epidemiology Research Branch, Intramural Research Program, National Institute of Mental Health, Bethesda, Maryland; 9 Nathan S. Kline Institute for Psychiatric Research, Orangeburg, New York

## Abstract

**Importance:**

Screening youths for mental disorders may assist in prevention, promote early identification, and be associated with reduced related lifetime impairment and distress.

**Objective:**

To assess parents’ and caregivers’ comfort with and preferences for pediatric mental health screening and factors associated with these preferences.

**Design, Setting, and Participants:**

This survey study used an online survey available from July 11 to 14, 2021, through Prolific Academic. Analyses were conducted from November 2021 to November 2022. The survey was administered to English-speaking parents and caregivers in the US, UK, Canada, and 16 other countries who were aged 21 years or older and had at least 1 child aged 5 to 21 years living at home.

**Main Outcomes and Measures:**

The main outcomes were parental preferences regarding content, implementation, and review of the findings of pediatric mental health screening. Parental comfort with screening topics was reported on a 6-point Likert scale with 6 indicating highest comfort. Mixed-effects logistic regression models were used to evaluate factors associated with parental comfort levels.

**Results:**

Of 1200 survey responses requested, data were collected from 1136 participants (94.7%). The final sample meeting the inclusion criteria comprised 972 parents and caregivers aged 21 to 65 years (mean [SD] age, 39.4 [6.9] years; 606 [62.3%] female). A total of 631 participants (64.9%) supported annual mental health screening for their child, and 872 (89.7%) preferred reviewing the screening results with professional staff (eg, physicians). Participants reported significantly decreased comfort with child self-report compared with parent-report screening assessments (*b* = −0.278; SE = 0.009; *P* < .001), although they were generally comfortable with both options. Despite slight variations based on country of residence, screening topic, and child’s age, participants were generally comfortable discussing all 21 screening topics on the survey. The greatest comfort was with sleep problems (mean [SE] score, 5.30 [0.03]); the least comfort was with firearms (mean [SE] score, 4.71 [0.05]), gender identity (mean [SE] score, 4.68 [0.05]), suicidality (mean [SE] score, 4.62 [0.05]), and substance use or abuse (mean [SE] score, 4.78 [0.05]).

**Conclusions and Relevance:**

In this survey study of parents and caregivers, parent-reported and child self-reported mental health screening in primary care settings was supported by the majority of the sample, although comfort levels differed according to various factors (eg, screening topic). Participants preferred to discuss screening results with professional health care staff. In addition to parental need for expert guidance, the study findings highlight the growing awareness of child mental health needs and the importance of addressing mental health concerns early via regular mental health screenings.

## Key Points

**Question:**

What are parents’ attitudes toward pediatric mental health screening in primary care settings?

**Findings:**

In this survey study of 972 English-speaking parents and caregivers in 19 countries, 93% of participants expressed interest in mental health screening of their children in primary care settings at regular intervals. Comfort with parent-report or child self-report options varied with the child’s age, reporting type, and topic assessed but were generally robust to the participants’ country of residence, with only slight variations.

**Meaning:**

The findings suggest that parents and caregivers are interested and willing to participate in mental health screening for their children in primary care settings.

## Teaser

This survey study conducted in 19 English-speaking countries investigates parents’ and caregivers’ comfort with and preferences for pediatric mental health screening assessments.

## Introduction

The growing prevalence and burden of mental health disorders in pediatric populations^1^ have made clear the need for improved detection of mental disorders.^2,3,4^ In particular, early identification of youth mental disorders via universal screening is an increasingly actionable solution with the potential to minimize the severity and progression of illness, mediate long-term impairment, and increase access to care,^5,6,7,8,9^ especially for common problems such as depression and anxiety.^10,11^ A growing amount of literature has drawn attention to primary health care settings^2,12,13^ as a natural point of integration, noting both the breadth of screenings already included in well-child visits and the reality that most mental health difficulties are first discussed with primary care practitioners (PCPs).^14,15,16^ Consistent with these notions, work has found that mental health referrals from PCPs are preferred and result in a higher follow-up rate compared with referrals from other parties.^17^ Accordingly, experts, health care systems, and local governments are increasingly promoting and building infrastructures to deploy mental health screening in primary care settings.^10,11,18,19,20,21,22,23,24^ However, it is also important to maximize the acceptability of screening. To date, much of the work around preferences for and acceptability of screening has focused on medical staff.^25,26,27,28^ In this study, we focused on the attitudes of parents and caregivers, which require careful attention to optimize implementation of screening.

Preliminary findings suggest that parents are generally supportive of pediatric mental health screening,^29,30,31,32,33^ although preferences exist. For example, studies have proposed that both parents and medical staff prefer mental health screening to occur during annual, routine visits.^27,34^ While some studies have found that parents prefer to review their child’s screening results with staff members who have medical expertise,^35,36^ there is variability based on the content of the screening instrument.^33^

Although 1 study showed relatively high acceptance rates of screening instrument topics (75%-85%), certain topics had significantly lower acceptance rates (eg, 50.4%), and percentages of acceptance appeared to substantially differ according to the topic.^33^ Attitudes toward a growing number of mental health topics are being assessed in the literature (eg, suicidality,^28,33,37,38^ substance use,^27,33^ firearms,^29,33,39^ depression,^25,28,33,40^ attention-deficit/hyperactivity disorder,^25,40^ anxiety,^25,40^ and gender identity^32^) although typically in isolation of one another,^27,28,32,37,41^ precluding a comprehensive picture.^25,29,33,40^ Additionally, previous studies^25,27,28,29,32,37,38,40,41^ often focused on the attitudes of patients and medical staff rather than parents and caregivers; they rarely studied the impact of the report option for the topic in question (eg, parent-report or a child self-report questionnaire). This is important because understanding parents’ comfort levels with mental health topics is essential for the development of effective screening procedures. In addition, parental acceptance of screening is likely to differ depending on whether they or their child is having the conversation.^35^ Furthermore, some researchers have identified the limits of solely relying on parent comments as a proxy for different types of problematic behavior in a child.^42,43,44^ Prior studies suggest that an individual’s country of residence may influence perceptions of mental disorders, in part due to cultural differences related to stigma and knowledge about resources for mental health.^45,46^ There is limited knowledge regarding parents’ comfort levels and preferences toward screening methods and content across international samples.

The present study examined comfort levels and preferences of parents and caregivers toward mental health screening in pediatric primary care settings using a novel survey that incorporated previous questionnaires and research along with input from experts and was administered to parents and caregivers from different English-speaking countries to assess general views of pediatric screening, methods, content, and report options (parent-report vs child self-report). Preferences were compared across countries to examine factors associated with parental preferences, comfort levels, and acceptability.

## Methods

### Participant Recruitment

Data for this survey study were collected from July 11 to 14, 2021, through Prolific Academic,^47^ an online, crowdsourced survey recruitment service open to participants aged 18 years or older and available in most Organization for Economic Co-operation and Development countries. Prolific Academic participants have been shown to be more sociodemographically diverse and provide higher-quality data compared with participants of similar data collection platforms.^48^ We requested samples from the US, the UK, Canada, and 16 other European and/or English-speaking countries (Australia, Austria, Belgium, Czech Republic, Denmark, France, Germany, Greece, Hungary, Ireland, Israel, Italy, the Netherlands, New Zealand, Poland, and Spain), which were grouped due to an insufficient number of parent samples on the platform. Prolific Academic participants were required to be fluent in English, be a parent or caregiver to 1 or more children (aged 5-21 years), and report about their oldest child in the study age range still living at home. There were no additional inclusion or exclusion criteria. Participants received $3 as compensation for a 15-minute survey. All data on Prolific Academic were collected anonymously after participants agreed to the terms of service; therefore, no additional informed consent was required. Approval and oversight were provided by the Advarra institutional review board. We followed the American Association for Public Opinion Research (AAPOR) reporting guideline.

### Study Design and Measures

The survey used for the present study was based on feedback from PCPs and mental health experts and from extensive reviews of prior studies exploring attitudes and preferences toward and/or barriers to mental health screening in youth populations (eTable in Supplement 1). The survey included 5 parts: background and demographics, willingness to discuss mental health, screening administration method, screening benefits and feedback, and parental comfort with screening topics (eAppendix in Supplement 1).

#### Background and Demographics

Participants were asked about their own and their child’s age, race and ethnicity, and gender identity. Race and ethnicity were included in the study to produce descriptive statistics of the sample characteristics; categories included American Indian/Alaska Native, Black/African American, Caribbean, East Asian/Pacific Islander, Latino/Latina/Latinx or Hispanic, Middle Eastern/North African, South/Southeast Asian, and White, with additional options for those who preferred to not answer, whose race or ethnicity was not listed, or who identified as 2 or more races and ethnicities. Additional questions addressed the family’s history of mental illness and frequency of physician visits.

#### Willingness to Discuss Mental Health

Respondents were asked to rate their agreement with 15 statements about mental health and learning disorders on a 6-point Likert scale (disagree [1] to agree [6]). The 15 statements assessed willingness for discussions (eg, “I am willing/able to discuss mental health with my child,” “I am willing/able to talk about my child’s learning difficulties with my family”) and perceptions of mental health (eg, “It should be equally easy to talk about both mental health and physical health”).

#### Screening Administration Method

Participants were asked 7 questions to assess their preferred mental health screening setting. One item queried the desired frequency of screening (monthly, quarterly, annually, or never). Five items assessed the preferred screening setting (eg, in the health care office, at the annual well-child visit only, or at home during a telehealth visit) on a 6-point Likert scale (disagree [1] to agree [6]). One multiselection checkbox item assessed participants’ preference regarding with which staff member (physician, nurse, other health care practitioner, office staff, social worker, psychologist, counselor, teacher, or other) they would like to discuss their child’s mental health issues.

#### Screening Benefits and Feedback

Four items assessed participants’ opinions regarding the possible benefits of mental health screening. The listed benefits were “early detection of problems,” “early intervention,” “learning more about my child,” and “other.” Participants rated their agreement with each benefit on a 6-point Likert scale (disagree [1] to agree [6]) and then were offered a free-response option to suggest additional benefits. Four items assessed participants’ preferences toward who completes the screening assessment and their preference for receiving results and feedback.

#### Parental Comfort With Screening Topics

Participants’ comfort levels with 21 topics were assessed as a parent-report option and as a child self-report option. Topics included depression, autism, suicidality, neurodevelopmental disorders, firearms, gender identity, and social media use. Comfort levels were rated on a 6-point Likert scale, with 6 indicating highest comfort.

### Statistical Analysis

Analyses were conducted from November 2021 to November 2022. Statistical analyses were conducted using R, version 2022.02.2 + 485 (packages lme4^49^ and stats^50^) (R Project for Statistical Computing). Descriptive statistics were determined for survey sections prior to more advanced analyses being conducted. For all analyses, a 2-sided statistical significance cutoff of *P* < .05 was applied. Benjamini-Hochberg correction^51^ was applied as appropriate. Linear and mixed-effects multivariate regression models were conducted to explore whether certain variables or interactions of variables were associated with parental comfort levels as a random variable.

## Results

### Sample Characteristics

Of 1200 survey responses requested, data were collected from 1136 participants (94.7%). Thirty-five of the 1136 participants (3.1%) completed only 70% to 85% of the survey. We excluded 164 participants, 22 of whom did not report their child’s age and 142 of whom had children outside the age range of our inclusion criteria. The final sample consisted of 972 parents and caregivers aged 21 years or older (mean [SD] age, 39.4 [6.9] years; range, 21 to 65 years); 606 (62.3%) were female, 356 (36.6%) were male, 4 (0.4%) were nonbinary, 1 (0.1%) was transgender female, 2 (0.2%) identified as other gender, and 3 (0.3%) did not report gender. Two participants (0.2%) were American Indian/Alaska Native; 51 (5.2%), Black/African American; none, Caribbean; 37 (3.8%), East Asian/Pacific Islander; 19 (2.0%), Latino/Latina/Latinx or Hispanic; 4 (0.4%), Middle Eastern/North African; 39 (4.0%), South/Southeast Asian; 766 (78.8%), White; and 35 (3.6%), 2 or more races and/or ethnicities; 6 (0.6%) preferred not to answer, and 13 (1.3%) indicated their race or ethnicity was not listed. Participants were grouped by their country of residence to ensure adequate power for across-country analyses: 265 (27.2%) were from the US, 282 (29.0%) from the UK, 171 (17.6%) from Canada, and 254 (26.1%) from other countries. Children were between the ages of 5 and 21 years (mean [SD] age: overall, 11.1 [4.3] years; US, 11.3 [4.2] years; UK, 10.7 [4.4] years; Canada, 11.4 [4.3] years; other countries, 11.1 [4.4] years). The US, UK, and Canada samples were predominantly female (174 [65.7%], 227 [80.5%], 85 [49.7%], respectively); the sample from the other countries was predominantly male (132 [52.0%]). The sample from the other countries included parents from 16 different countries. Demographic data are provided in the Table.

**Table.** Sample Characteristics

Characteristic	Participants^a^				
	Total (N = 972)	US (n = 265)	UK (n = 282)	Canada (n = 171)	Other countries (n = 254)
**Parent or caregiver**					
Age, mean (SD), y	39.4 (6.9)	38.1 (6.8)	39.0 (7.0)	39.9 (6.6)	41.9 (19.6)
Gender					
Female	606 (62.3)	174 (65.7)	227 (80.5)	85 (49.7)	120 (47.2)
Male	356 (36.6)	89 (33.6)	51 (18.1)	84 (49.1)	132 (52.0)
Nonbinary	4 (0.4)	2 (0.8)	0	0	2 (0.8)
Transgender female	1 (0.1)	0	0	1 (0.6)	0
Transgender male	0	0	0	0	0
Other	2 (0.2)	0	1 (0.4)	1 (0.6)	0
Prefer not to answer	3 (0.3)	0	3 (1.1)	0	0
Race and ethnicity					
American Indian/Alaska Native	2 (0.2)	1 (0.4)	1 (0.4)	0	0
Black/African American	51 (5.2)	30 (11.3)	4 (1.4)	12 (7.0)	5 (2.0)
Caribbean	0	0	0	0	0
East Asian/Pacific Islander	37 (3.8)	6 (2.3)	2 (0.7)	21 (12.3)	8 (3.1)
Latino/Latina/Latinx or Hispanic	19 (2.0)	7 (2.6)	1 (0.4)	5 (2.9)	6 (2.4)
Middle Eastern/North African	4 (0.4)	0	1 (0.4)	0	3 (1.2)
South/Southeast Asian	39 (4.0)	3 (1.1)	13 (4.6)	13 (7.6)	10 (3.9)
White	766 (78.8)	201 (75.8)	247 (87.6)	108 (63.2)	210 (82.7)
≥2 Races and/or ethnicities	35 (3.6)	15 (5.7)	4 (1.4)	9 (5.3)	7 (2.8)
Prefer not to answer	6 (0.6)	0	3 (1.1)	2 (1.2)	1 (0.4)
Race or ethnicity not listed	13 (1.3)	2 (0.8)	6 (2.1)	1 (0.7)	4 (1.6)
**Child**					
Age, mean (SD), y	11.1 (4.3)	11.3 (4.2)	10.7 (4.4)	11.4 (4.3)	11.1 (4.4)
Gender					
Female	442 (45.5)	124 (46.8)	126 (44.7)	77 (45.0)	115 (45.3)
Male	509 (52.4)	134 (50.6)	149 (52.8)	92 (53.8)	134 (52.8)
Nonbinary	9 (0.9)	5 (1.9)	1 (0.4)	1 (0.6)	2 (0.8)
Transgender female	2 (0.2)	1 (0.4)	0	0	1 (0.4)
Transgender male	1 (0.1)	0	1 (0.4)	0	0
Other	2 (0.2)	0	1 (0.4)	0	1 (0.4)
Prefer not to answer	7 (0.7)	1 (0.4)	4 (1.4)	1 (0.6)	1 (0.4)
Race and ethnicity					
American Indian/Alaska Native	1 (0.1)	1 (0.4)	0	0	0
Black/African American	45 (4.6)	27 (10.2)	5 (1.8)	9 (5.3)	4 (1.6)
Caribbean	0	0	0	0	0
East Asian/Pacific Islander	33 (3.5)	5 (1.9)	3 (1.1)	20 (11.7)	5 (2.0)
Latino/Latina/Latinx or Hispanic	16 (1.6)	6 (2.3)	0	4 (2.3)	4 (2.3)
Middle Eastern/North African	3 (0.3)	0	0	0	3 (1.1)
South/Southeast Asian	35 (3.6)	5 (1.9)	11 (3.9)	11 (6.4)	8 (3.1)
White	732 (75.3)	182 (68.7)	241 (85.5)	103 (60.2)	206 (81.1)
≥2 Races and/or ethnicities	76 (7.8)	38 (14.3)	11 (4.3)	18 (10.5)	11 (4.3)
Prefer not to answer	6 (0.6)	1 (0.4)	3 (1.1)	2 (1.2)	1 (0.4)
Race or ethnicity not listed	25 (2.6)	10 (3.8)	10 (3.5)	4 (2.3)	10 (3.9)
**Family**					
Location					
Large metropolitan area	134 (13.8)	25 (9.4)	27 (9.6)	36 (21.1)	46 (18.1)
Metropolitan area	133 (13.7)	38 (14.3)	27 (9.6)	38 (22.2)	30 (11.8)
Medium-size urban area	142 (14.6)	23 (8.7)	49 (17.4)	26 (15.2)	44 (17.3)
Small metropolitan area	135 (13.9)	27 (10.2)	44 (15.6)	22 (12.9)	42 (16.5)
Large suburban area	134 (13.8)	52 (19.6)	35 (12.4)	21 (12.3)	26 (10.2)
Small suburban area	172 (17.7)	58 (21.9)	56 (19.9)	20 (11.7)	38 (15.0)
Rural area	122 (12.6)	42 (15.8)	44 (15.6)	8 (4.7)	28 (11.0)
Annual income, US $, thousands					
<25	101 (10.4)	35 (13.2)	41 (14.5)	2 (1.2)	23 (9.1)
25 to <50	217 (22.3)	46 (17.4)	87 (30.9)	16 (9.4)	68 (26.8)
50 to <75	213 (21.9)	68 (25.7)	63 (22.3)	29 (17.0)	53 (20.9)
75 to <100	135 (13.9)	37 (14.0)	37 (14.0)	27 (15.8)	35 (13.8)
100 to <125	98 (10.1)	22 (8.3)	22 (8.3)	29 (17.0)	23 (9.1)
125 to <150	70 (7.2)	24 (9.1)	24 (9.1)	23 (13.5)	16 (6.3)
150 to <200	60 (6.2)	16 (6.0)	16 (6.0)	22 (12.9)	13 (5.1)
200 to <250	29 (3.0)	6 (2.3)	6 (2.3)	12 (7.0)	8 (3.1)
250 to <300	13 (1.3)	7 (2.6)	7 (2.6)	3 (1.8)	2 (0.8)
≥300	2 (0.2)	1 (0.4)	1 (0.4)	1 (0.6)	0
Prefer not to answer	34 (3.5)	3 (1.1)	3 (1.1)	7 (4.1)	13 (5.1)

^a^ Data are presented as the number (percentage) of participants unless otherwise indicated.

### Survey Results

A total of 895 participants (92.1%) reported that they wanted their child screened for mental health problems at regular intervals. Annual screening was preferred by 631 participants (64.9%), followed by quarterly screening (226 [23.3%]) (eFigure 1 in Supplement 1).

**Figure 1.**
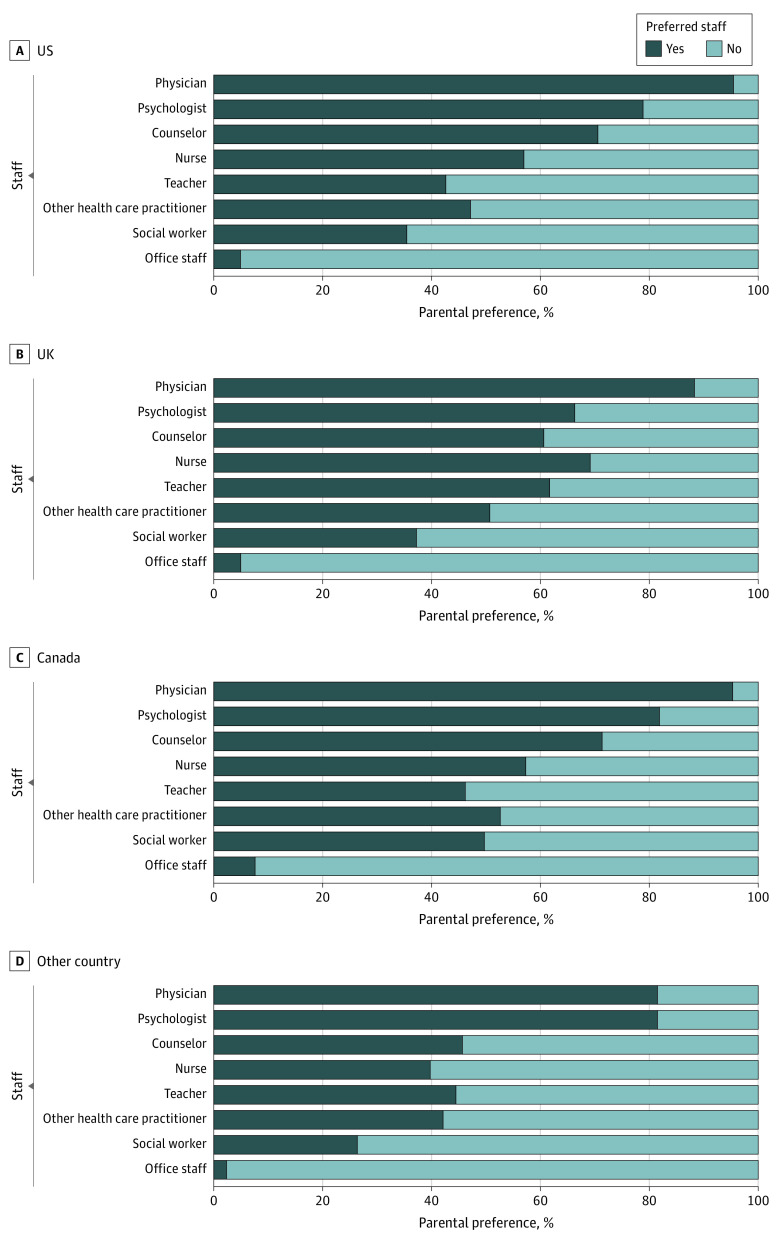
Parental Preferences for Reviewing Screening Findings With Medical Staff Members Parents and caregivers were asked whether they were comfortable (“yes”) or not comfortable (“no”) with each type of staff member.

Across the entire sample, participants were most willing (yes or no) to speak with physicians (872 [89.7%]), followed by psychologists (743 [76.4%]). Only 46 participants (4.8%) were willing to discuss the screening results with general office staff (Figure 1). Figure 1 shows the slight variances that were observed by country. When looking at specific data sources, the most notable variations across country samples were participants’ willingness to speak with teachers (US, 113 [42.6%]; UK, 174 [61.7%]; Canada, 79 [46.2%]; other countries, 113 [44.5%]) and social workers (US, 94 [35.5%]; UK, 105 [37.2%]; Canada, 85 [49.7%]; other countries, 67 [26.4%]). Over 65% of participants from each country sample expressed a willingness to talk about their child’s mental health with physicians (US, 253 [95.5%]; UK, 249 [88.3%]; Canada, 163 [95.3%]; other countries, 207 [81.5%]) and psychologists (US, 209 [78.9%]; UK, 187 [66.3%]; Canada, 140 [81.9%]; other countries, 207 [81.5%]), whereas less than 8% of participants from each country were willing to review screening assessment results with general office staff (US, 13 [4.9%]; UK, 14 [5.0%]; Canada, 13 [7.6%]; other countries, 6 [2.4%]).

**Figure 2.**
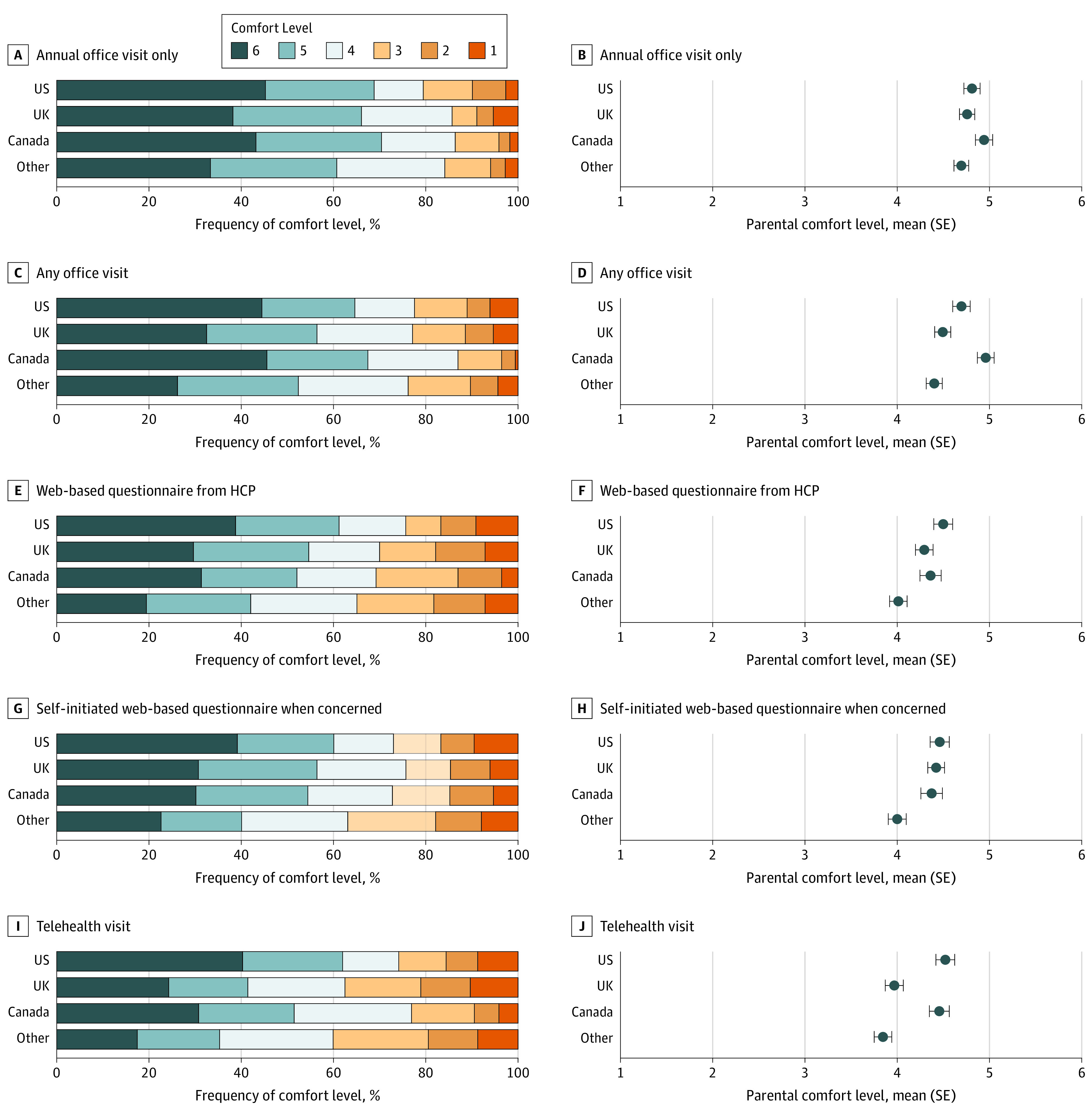
Parental Comfort Levels for 5 Screening Administration Settings A comfort level of 6 indicates greatest comfort. HCP indicates health care practitioner.

Screening contexts and topics were assessed on a 6-point Likert scale. Figure 2 shows participants’ comfort level for 5 screening contexts. Participants indicated lower comfort with screening at home compared with in the health care office (*b* = −0.44; SE, 0.04; *P* < .001). Participants’ country of residence and the child’s age were accounted for in the regressions. Compared with the US sample, participants from other countries (*b* = −0.40; SE, 0.06; *P* < .001) and from the UK (*b* = −0.20; SE, 0.06; *P* < .001) reported decreased levels of comfort; no statistically significant difference was reported by participants from Canada. Higher comfort levels were associated with older children (*b* = 0.02; SE, 0.01; *P* < .001).

A regression model compared the 3 at-home administration contexts: self-guided web-based assessments (mean [SE] Likert score, 4.31 [0.05]), telehealth visits (mean [SE] score, 4.17 [0.05]), and practitioner-guided web-based assessments (mean [SE] score, 4.29 [0.05]). Two in-office administration contexts were also compared: annual visits (4.79 [0.04]) and any visit (4.61 [0.05]). Compared with the context with the highest comfort level (in office, annual visit), the model found significantly lower comfort for the 4 other contexts: in office, any visit (*b* = −0.18; SE, 0.05; *P* < .001); at home, self-guided web-based assessment (*b* = −0.47; SE, 0.05; *P* < .001); at home, practitioner-guided web-based assessment (*b* = −0.50; SE, 0.05; *P* < .001); and at home, telehealth visit (*b* = −0.62; SE, 0.05; *P* < .001).

A mixed-effects regression model that included the parent’s country of residence, the child’s age, each screening topic, and the respondent option found that participants reported significantly decreased comfort with child self-report compared with parent-report screening assessments (*b* = −0.278; SE, 0.009; *P* < .001). Furthermore, for every 1-year increase in the child’s age, parental comfort levels increased (*b* = 0.035; SE, 0.008; *P* < .001). Mixed-effects regression models for each of the topics found that participants were significantly more comfortable with parent-report compared with child self-report for all 21 topics (*b* = −0.10 [SE, 0.03]; *P* = .002 to −0.49 [SE, 0.05]; *P* < .001). All parent-report vs child self-report findings remained significant after corrections using the Benjamini-Hochberg method^51^ (*b* = −0.10 [SE, 0.03]; *P* = .002 to −0.49 [SE, 0.05]; *P* < .001).

**Figure 3.**
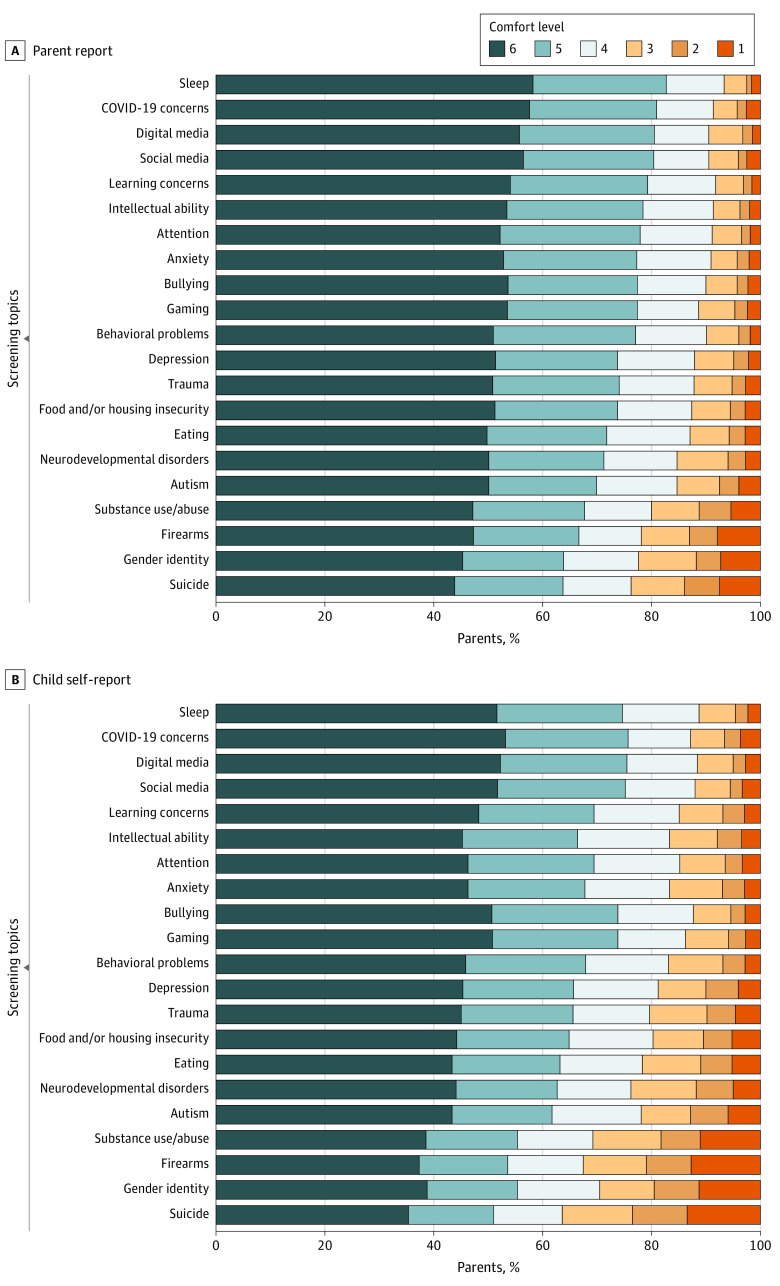
Distribution of Parental Comfort Levels of Screening Topics by Report Option Mixed-effects regression models with Benjamini-Hochberg corrections. A comfort level of 6 indicates greatest comfort.

**Figure 4.**
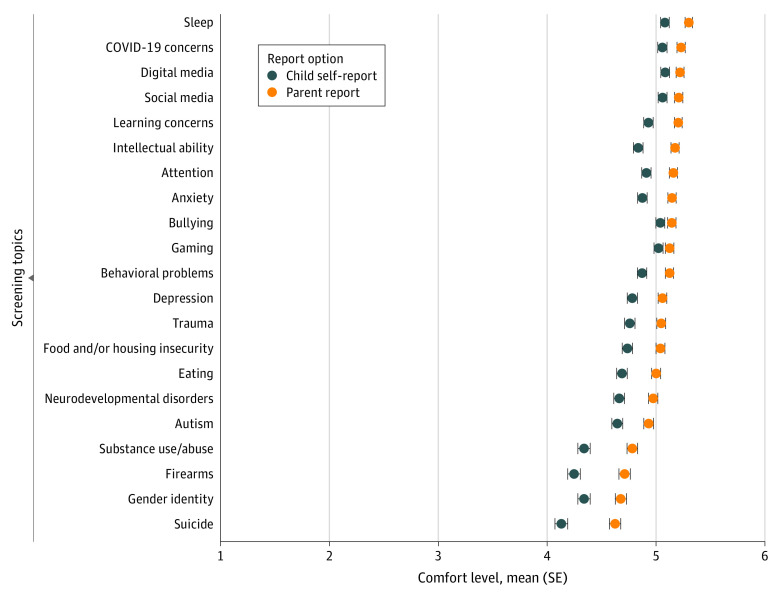
Mean Parental Comfort Levels of Screening Topics by Report Option Mixed-effects regression models with Benjamini-Hochberg corrections. A comfort level of 6 indicates greatest comfort.

As shown in Figure 3 and Figure 4, parental comfort levels with 21 screening topics differed by the topic and by the report option (parent-report vs child self-report). Mean (SE) parental comfort levels on a 6-point Likert scale ranged from 4.62 (0.05) to 5.30 (0.03). Topics on which participants were most comfortable reporting included child sleep problems (mean [SE] score, 5.30 [0.03]), COVID-19 concerns (mean [SE] score, 5.23 [0.04]), digital media use (mean [SE] score, 5.22 [0.04]), social media use (mean [SE] score, 5.21 [0.04]), and learning concerns (mean [SE] score, 5.20 [0.04]). Participants were the least comfortable reporting on their child’s experience with substance use or abuse (mean [SE] score, 4.78 [0.05]), firearms (mean [SE] score, 4.71 [0.05]), gender identity (mean [SE] score, 4.68 [0.05]), and suicidal ideation (mean [SE] score, 4.62 [0.05]). For child self-report, mean (SE) parental comfort levels on a 6-point Likert scale ranged from 4.13 (0.06) to 5.08 (0.04). Participants were the most comfortable with their child reporting on their digital media use (mean [SE] score, 5.08 [0.04]), sleep problems (mean [SE] score, 5.08 [0.04]), social media use (mean [SE] score, 5.06 [0.04]), COVID-19 concerns (mean [SE] score, 5.06 [0.04]), and bullying (mean [SE] score, 5.04 [0.04]). The child self-report topics that participants were the least comfortable with were gender identity (mean [SE] score, 4.34 [0.06]), substance use or abuse (mean [SE] score, 4.34 [0.06]), firearms (mean [SE] score, 4.25 [0.06]), and suicidal ideation (mean [SE] score, 4.13 [0.06]). Some variations were observed in parental comfort with specific topics across country samples (eFigure 2 in Supplement 1).

Parental comfort levels for child self-reporting were correlated with their child’s age for all 21 topics (*b* = 0.10 [SE, 0.03]; *P* = .002 to 0.27 [SE, 0.03]; *P* < .001) (eFigure 3 in Supplement 1). Parental comfort levels for 11 of the 21 parent-reporting topics (52.4%) were correlated with the child’s age (substance use or abuse, suicidal ideation, firearms, gender identity, depression, autism, COVID-19 concerns, gaming, social media use, digital media use, and food and/or housing insecurities) (*b* = 0.06 [SE, 0.03]; *P* = .047 to 0.12 [SE, 0.03]; *P* < .001). When controlling for the parent’s age, these findings remained relatively consistent, with sleep problems, depression, and anxiety also being significantly associated with the child’s age.

Over 90% of participants agreed (≥4 on 6-point Likert scale) that “early detection of problems,” “early intervention,” and “to learn more about my child” were benefits from mental health screening. Other reported benefits included better “access [to] mental health resources,” “awareness of signs to watch for,” ability “to accommodate/support my child,” “management of symptoms,” and “prevention of problems.”

## Discussion

The present study found that a majority of parents and caregivers were comfortable having their child screened for all mental health topics probed in the survey. However, several preferences were observed. First, participants expressed a preference for carrying out screenings on an annual basis—a model that fits well with that of general medical screenings in the primary care setting. Second, participants favored completing the screening assessment in health care offices rather than at home, although comfort levels for at-home screening were still relatively high. Third, there was a preference for having physicians and psychologists provide the interpretation of the findings, with notably lower comfort levels for reviewing results with social workers, general office staff, or teachers. Regarding screening content, we found that participants’ comfort was dependent on screening content and report option (parent-report vs child self-report). Participants were generally comfortable with all 21 screening topics assessed in the present study (mean [SE] Likert score range, 4.13 [0.06] to 5.30 [0.03]). Although 4 topics—substance use or abuse, firearms, gender identity, and suicidal ideation—had consistently lower comfort levels, participants reported relatively high comfort with these topics (mean [SD] score range, 4.13 [0.06] to 4.78 [0.05]). Parents and caregivers preferred to complete the screening assessments themselves, although they were still relatively comfortable with allowing their child to complete a self-report assessment, with their comfort increasing with the child’s age. Finally, it is worth noting that our findings were not dependent on country, although some variation in overall comfort levels across countries was present.

Beyond supporting the acceptability of pediatric mental health screening in primary care settings to parents, the present work also suggests potential areas for optimization in future efforts. First, our findings suggest that home-based screenings can minimize workflow interruptions and time costs associated with screening^27,28,32,37,52^ and are an acceptable solution for many parents. As web-based screening assessments become more widely available and are integrated into electronic health record systems, health care offices may consider this route of administration. This may also allow for increased frequency of screenings, as certain mental illnesses are known to fluctuate by season,^53^ suggesting that annual screening may not be sufficient for all disorders. Future work should explore the best times of the year to screen youths and whether home-based screening can better capture some of these fluctuations. Second, parents and caregivers appeared less comfortable with direct screening of their children than via their own report. Although less concerning for the detection of externalizing disorders, such as attention-deficit/hyperactivity disorder, this can be problematic for the detection of internalizing disorders, such as anxiety and depression. Additional analyses aligned with the US Preventive Services Task Force’s recommendation of child self-report screening beginning at age 12 years,^10^ although future research is needed. Some participants expressed decreased comfort with assessment of key topics related to risk of harm (eg, suicidal ideation, substance use, and firearms). Increased efforts toward the education of parents about the potential benefits and risks of screening may help to increase comfort levels for more comprehensive screening processes.

Our finding of a preference for interpretation of screening assessment results by medical professionals may have implications for efforts focused on school-based screening. In particular, it suggests that school-based efforts may benefit from either having a medical or psychological professional on site to have these conversations periodically or transferring the screening results to the child’s primary care clinicians for discussion with families. Implementing screenings in both primary care and school-based settings may then address concerns of time demands and other barriers from screening staff^24^ in addition to improving identification and detection. Future work is needed to better understand how to support screening practices in these settings regarding education and training, management, and finances, with consideration of common business models and workflows of PCPs.

### Limitations

Limitations of this study include a requirement that participants be fluent in English and have knowledge of and access to Prolific Academic, an online resource. These inclusion criteria prevented parents and caregivers with limited English skills and/or access to the English-based internet site from participating in the study. Inclusion of data from multiple countries suggests some level of generalizability of findings, although it does not exclude potential bias. To our knowledge, current research has not yet addressed cultural and geographic differences in openness to screen, interpret, and take action on pediatric mental health problems and behaviors. Previous studies have suggested that socioeconomic and demographic factors (eg, race and ethnicity and annual household income) may affect results.^54,55,56^ Interestingly, the present study did not find an association between these factors and parental comfort levels; however, given the lack of racial and ethnic diversity in the present sample, this requires further study. A systematic review of previous studies noted a variety of changes to family life as a result of the COVID-19 pandemic.^57^ Thus, another potential limitation arises from the pandemic occurring simultaneously with this survey, which may have influenced participation rates and responses.

## Conclusions

In this survey study of parents and caregivers, there was cross-national parent and caregiver acceptability for mental health screening of their offspring, with preferences for follow-up with experts who can facilitate further evaluation or treatment. This study suggests the need to engage both professionals and the public who may benefit from screening and some of the key factors (eg, screening topics, child age, country of residence, and report option) that may enhance the development of future programs to detect and intervene in mental disorders in youths.

## Notes

Supplement 1. **eAppendix.** Pediatric Screening Survey **eTable.** Key References for Curation of the Pediatric Screening Survey **eFigure 1.** Parents’ Willingness to Discuss Mental Health and Learning Disorders **eFigure 2**. Mean Parental Comfort Levels of Screening Topics by Country **eFigure 3.** Correlation of Child’s Age and Parents’ Comfort With Screening Topics by Report Option

Supplement 2.
Data Sharing Statement
