# Supplementary material for: Parental Preferences for Mental Health Screening of Youths From a Multinational Survey
Source: JAMA Netw Open. 2023 Jun 20;6(6):e2318892. doi: 10.1001/jamanetworkopen.2023.18892 (PMC10282888; doi:10.1001/jamanetworkopen.2023.18892)
Supplement: Supplement 1. — eAppendix. Pediatric Screening Survey eTable. Key References for Curation of the Pediatric Screening Survey eFigure 1. Parents’ Willingness to Discuss Mental Health and Learning Disorders eFigure 2. Mean Parental Comfort Levels of Screening Topics by Country eFigure 3. Correlation of Child’s Age and Parents’ Comfort With Screening Topics by Report Option [file jamanetwopen-e2318892-s001.pdf]

Supplementary Online Content

Kass M, Alexander L, Moskowitz K, et al. Parental preferences for mental health screening of youths from a multinational survey. *JAMA Netw Open*. 2023;6(6):e2318892. doi:10.1001/jamanetworkopen.2023.18892

eAppendix. Pediatric Screening Survey
eTable. Key References for Curation of the Pediatric Screening Survey
eFigure 1. Parents' Willingness to Discuss Mental Health and Learning Disorders
eFigure 2. Mean Parental Comfort Levels of Screening Topics by Country
eFigure 3. Correlation of Child's Age and Parents' Comfort With Screening Topics by Report Option

This supplementary material has been provided by the authors to give readers additional information about their work.

© 2023 Kass M et al. *JAMA Network Open*.

eAppendix. Pediatric Screening Survey.

This survey will ask your opinion about screening your child for mental health problems in the primary care setting. First you will be asked to complete some background information about you and your child.

Prolific Academic ID	______________________________
Country of Residence	○ United States<br>○ United Kingdom<br>○ Canada<br>○ Other
Please specify:	______________________________
State or Province:	______________________________

BACKGROUND

What is your age?	______________________________
What is your gender identity?	○ Male<br>○ Female<br>○ Non-binary<br>○ Transgender male<br>○ Transgender female<br>○ Other<br>○ Prefer not to answer
Which of these categories best describes you? Please select all that apply.	• White<br>• Black / African American<br>• Latino / Latina / Latinx or Hispanic<br>• American Indian or Alaska Native<br>• East Asian or Pacific Islander<br>• South or Southeast Asian<br>• Middle Eastern or North African<br>• Caribbean<br>• A race, ethnicity, or origin not listed<br>• Prefer not to answer
How many children do you have?	______________________________

Please answer the background information below about your oldest child living at home:

© 2023 Kass M et al. *JAMA Network Open*.

What is your child's age?	_____________________________________
What is your child's gender identity?	○ Male<br>○ Female<br>○ Non-binary<br>○ Transgender male<br>○ Transgender female<br>○ Other<br>○ Prefer not to answer
Which of these categories best describes you? Please select all that apply.	• White<br>• Black / African American<br>• Latino / Latina / Latinx or Hispanic<br>• American Indian or Alaska Native<br>• East Asian or Pacific Islander<br>• South or Southeast Asian<br>• Middle Eastern or North African<br>• Caribbean<br>• A race, ethnicity, or origin not listed<br>• Prefer not to answer
Which best describes the area in which your child lives?	○ Large metropolitan area (population greater than 1.5 million)<br>○ Metropolitan area (population 500,000-1.5 million)<br>○ Medium-size urban area (population 200,000-500,000)<br>○ Small metropolitan area (population 50,000-200,000)<br>○ Large suburban area (population 25,000-50,000)<br>○ Small suburban area (population 5,000-25,000)<br>○ Rural area (population less then 5,000)
What is the highest level of education YOU completed?	○ Some grade school<br>○ Some high school<br>○ High school diploma or GED<br>○ Some college or 2-year degree<br>○ 4-year college graduate<br>○ Some school beyond college<br>○ Graduate or professional degree (e.g., MA, MS, MBA, PhD, EdD, MD, DDS, JD, DVM)
Please choose the category that most closely matches YOUR occupation. If you are not currently working for pay please use your most recent occupation.	○ Day laborer, janitor, house cleaner, farm worker, food counter sales, food preparation worker, busboy.<br>○ Garbage collector, short-order cook, cab driver, shoe sales, assembly line workers, masons, baggage porter.<br>○ Painter, skilled construction trade, sales clerk, truck driver, cook, sales counter or general office clerk.<br>○ Automobile mechanic, typist, locksmith, farmer, carpenter, receptionist, construction laborer, hairdresser.

© 2023 Kass M et al. *JAMA Network Open*.

	○ Machinist, musician, bookkeeper, secretary, insurance sales, cabinet maker, personnel specialist, welder.<br>○ Supervisor, librarian, aircraft mechanic, artist and artisan, electrician, administrator, military enlisted personnel, buyer.<br>○ Nurse, skilled technician, medical technician, counselor, manager, police and fire personnel, financial manager, physical, occupational, speech therapist.<br>○ Mechanical, nuclear, and electrical engineer, educational administrator, veterinarian, military officer, elementary, high school and special education teacher.<br>○ Physician, attorney, professor, chemical and aerospace engineer, judge, CEO, senior manager, public official, psychologist, pharmacist, accountant.
Does your child have a second parent or caregiver?	○ Yes<br>○ No
What is the highest level of education your child's other main parent/caregiver completed?	○ Some grade school<br>○ Some high school<br>○ High school diploma or GED<br>○ Some college or 2-year degree<br>○ 4-year college graduate<br>○ Some school beyond college\<br>○ Graduate or professional degree (e.g., MA, MS, MBA, PhD, EdD, MD, DDS, JD, DVM)
Please choose the category that most closely matches your child's other main parent/caregiver's occupation. If the other caregiver is not currently working for pay please use your most recent occupation.	○ Day laborer, janitor, house cleaner, farm worker, food counter sales, food preparation worker, busboy.<br>○ Garbage collector, short-order cook, cab driver, shoe sales, assembly line workers, masons, baggage porter.<br>○ Painter, skilled construction trade, sales clerk, truck driver, cook, sales counter or general office clerk.<br>○ Automobile mechanic, typist, locksmith, farmer, carpenter, receptionist, construction laborer, hairdresser.<br>○ Machinist, musician, bookkeeper, secretary, insurance sales, cabinet maker, personnel specialist, welder.<br>○ Supervisor, librarian, aircraft mechanic, artist and artisan, electrician, administrator, military enlisted personnel, buyer.<br>○ Nurse, skilled technician, medical technician, counselor, manager, police and fire personnel, financial manager, physical, occupational, speech therapist.<br>○ Mechanical, nuclear, and electrical engineer, educational administrator, veterinarian, military officer, elementary, high school and special education teacher.<br>○ Physician, attorney, professor, chemical and aerospace engineer, judge, CEO, senior manager, public official, psychologist, pharmacist, accountant.

© 2023 Kass M et al. *JAMA Network Open*.

What is your annual household income?	○ Less than $10,000<br>○ $10,000 to $19,999<br>○ $20,000 to $29,999<br>○ $30,000 to $39,999<br>○ $40,000 to $49,999<br>○ $50,000 to $59,999<br>○ $60,000 to $69,999<br>○ $70,000 to $79,999<br>○ $80,000 to $89,999<br>○ $90,000 to $99,999<br>○ $100,000 to $149,999<br>○ $150,000 or more<br>○ Choose not to disclose
How many people are members of your household?	________________________________
Is your child covered by health insurance?	○ Yes, military<br>○ Yes, employer-sponsored<br>○ Yes, individual<br>○ Yes, Medicare<br>○ Yes, Medicaid or medicaid supported program<br>○ Yes, CHIP<br>○ Yes, other<br>○ I don't know<br>○ No
Has a health or education professional ever said that someone in your family has any of the following health conditions (check all that apply)?	• Depression<br>• Anxiety<br>• Problems with alcohol or drugs<br>• Post-traumatic Stress Disorder (PTSD)<br>• Attention Deficit Hyperactivity Disorder (ADHD)<br>• Intellectual disability<br>• Autism Spectrum Disorder<br>• Learning Disorder<br>• None of the Above<br>• Other
Please Specify.	________________________________
Has anyone in your child's family been told that they have a mental health or learning disorder (select all that apply)?	• Yes, biological mother<br>• Yes, biological father<br>• Yes, sibling<br>• No
Biological Mother Diagnosis (check all that apply):	• Depression<br>• Anxiety

	• Problems with alcohol or drugs<br>• Post-traumatic Stress Disorder (PTSD)<br>• Attention Deficit Hyperactivity Disorder (ADHD)<br>• Intellectual disability<br>• Autism Spectrum Disorder<br>• Learning Disorder<br>• Schizophrenia or Psychosis<br>• Other<br>• None of the Above<br>• Prefer not to answer
Please Specify.	___________________________________
Biological Father Diagnosis (check all that apply):	• Depression<br>• Anxiety<br>• Problems with alcohol or drugs<br>• Post-traumatic Stress Disorder (PTSD)<br>• Attention Deficit Hyperactivity Disorder (ADHD)<br>• Intellectual disability<br>• Autism Spectrum Disorder<br>• Learning Disorder<br>• Schizophrenia or Psychosis<br>• Other<br>• None of the Above<br>• Prefer not to answer
Sibling Diagnosis (check all that apply):	• Depression<br>• Anxiety<br>• Problems with alcohol or drugs<br>• Post-traumatic Stress Disorder (PTSD)<br>• Attention Deficit Hyperactivity Disorder (ADHD)<br>• Intellectual disability<br>• Autism Spectrum Disorder<br>• Learning Disorder<br>• Schizophrenia or Psychosis<br>• Other<br>• None of the Above<br>• Prefer not to answer
Does your child have a primary care medical provider such as a pediatrician, family doctor, nurse practitioner?	○ Yes<br>○ No
Does your child attend annual healthcare checkups?	○ Yes<br>○ No
On average how frequently does your child visit their health care provider (including checkups)?	○ None<br>○ 1-2 times per year

© 2023 Kass M et al. *JAMA Network Open*.

	○ 3-4 times per year<br>○ More than 4 times per year
How many emergency visits does your child have each year?	○ None<br>○ 1-2 times per year<br>○ 3-4 times per year<br>○ More than 4 times per year
Has your child received mental health care in the past 12 months?	○ Yes<br>○ No
Have you received mental health care in the past 12 months?	○ Yes<br>○ No

Please rate your agreement with each of the following statements below.

	1=Disagree	2	3	4	5	6=Agree
I am willing/able to talk about mental health.						
I am willing/able to talk about my personal mental health						
People are now more willing to talk about mental health						
People are more willing to talk about children's mental health						
I've seen celebrities talk about their own mental health						
Celebrities seem to talk more about their own mental health						
It should be equally easy to talk about both mental health and physical health						
I am willing/able to talk about my own child's mental health						
I am willing/able to talk about my own child's learning difficulties						
I am willing/able to talk about my mental health with my child						
I am willing/able to talk about my child's mental health with my family						
I am willing/able to talk about my child's learning difficulties with my family						
I am willing/able to talk about my child's mental health with my friends						
I am willing/able to talk about my child's learning difficulties with my friends						
There is a stigma associated with mental health in the United States.						

© 2023 Kass M et al. *JAMA Network Open*.

How often would you be comfortable having your child screened for potential mental health concerns:	○ Monthly<br>○ Quarterly<br>○ Annually<br>○ Never

I feel comfortable with mental health screenings in the following locations:

	1=Disagree	2	3	4	5	6=Agree
In healthcare office, at annual well-child visit only						
In healthcare office, no preference regarding visit type (e.g., well, sick)						
At home, with web based questionnaire from healthcare provider						
At home, self-initiated web based questionnaire (when you have a concern)						
At home, telehealth visit						

Screening my child is beneficial for:

	1=Disagree	2	3	4	5	6=Agree
Early detection of problems						
Early intervention						
Learning more about my child						
Other						

Please describe. ______________________________

Who will complete mental health screenings of your child (check all that apply)?	• You (parent)<br>• Your child<br>• Your child's health care provider<br>• Teacher/school<br>• Other

Please describe. ______________________________

What results do you want from mental health screening (check all that apply)?	• Information about my child<br>• Resources/parent training on how to help my child<br>• Services and referrals<br>• Other

Please describe. ______________________________

How do you prefer to receive results from your child's mental health screening?	○ In person/directly from a clinician<br>○ Emailed/mailed report from clinician

© 2023 Kass M et al. *JAMA Network Open*.

	○ Automated report with recommendations<br>○ Other
Please describe.	___________________________________
How would you like to receive feedback recommendations and educational materials (check all that apply):	• Websites<br>• Emails<br>• Handouts<br>• Educational videos<br>• Directly from a clinician
With whom are you willing/able to discuss mental health issues about your child? (check all that apply)	• Physician<br>• Nurse<br>• Other health care provider<br>• Office staff<br>• Social Worker<br>• Psychologist<br>• Counselor<br>• Teacher<br>• Other
Please specify:	___________________________________

I feel comfortable being asked about the following about my child:

	1=Disagree	2	3	4	5	6=Agree
Depression						
Anxiety						
Behavior Problems						
Substance Abuse						
Attention Abilities						
Intellectual Abilities						
Autism						
Suicidality						
Eating Behaviors						
Bullying						
Neurodevelopmental Disorders						
Firearms						
Gender Identity						
Learning Concerns						
Sleep Problems						
Food and Housing Insecurity						
Trauma						
Gaming						

© 2023 Kass M et al. *JAMA Network Open*.

Digital Media Use						
Social Media Use						
Concerns Related to Covid-19						

I feel comfortable with MY CHILD being asked about the following:

	1=Disagree	2	3	4	5	6=Agree
Depression						
Anxiety						
Behavior Problems						
Substance Abuse						
Attention Abilities						
Intellectual Abilities						
Autism						
Suicidality						
Eating Behaviors						
Bullying						
Neurodevelopmental Disorders						
Firearms						
Gender Identity						
Learning Concerns						
Sleep Problems						
Food and Housing Insecurity						
Trauma						
Gaming						
Digital Media Use						
Social Media Use						
Concerns Related to Covid-19						

© 2023 Kass M et al. *JAMA Network Open*.

eTable 1. Key References for Curation of the Pediatric Screening Survey.

Efficacy and Barriers of Implementing Pediatric Mental Health Screening		
Study Title	Sample Population	Full Citation
Screening for Adverse Childhood Experiences in Primary Care: A Quality Improvement Project.	Medical Staff	Bryant C, VanGraafeiland B. Screening for Adverse Childhood Experiences in Primary Care: A Quality Improvement Project. *J Pediatr Health Care*. 2020;34(2):122-127.
Use of the Pediatric Symptom Checklist to screen for psychosocial problems in pediatric primary care: a national feasibility study.	Children and Adolescents	Jellinek MS, Murphy JM, Little M, Pagano ME, Comer DM, Kelleher KJ. Use of the Pediatric Symptom Checklist to screen for psychosocial problems in pediatric primary care: a national feasibility study. *Arch Pediatr Adolesc Med*. 1999;153(3):254-260.
Integrating Suicide Risk Screening into Pediatric Ambulatory Subspecialty Care.	Patients (9- to 21-years)	Lois BH, Urban TH, Wong C, et al. Integrating Suicide Risk Screening into Pediatric Ambulatory Subspecialty Care. *Pediatr Qual Saf*. 2020;5(3):e310.
Barriers and facilitators affecting the implementation of substance use screening in primary care clinics: a qualitative study of patients, providers, and staff.	Medical Staff<br>Patients	McNeely J, Kumar PC, Rieckmann T, et al. Barriers and facilitators affecting the implementation of substance use screening in primary care clinics: a qualitative study of patients, providers, and staff. *Addict Sci Clin Pract*. 2018;13(1):8.
Screening for behavioral health problems in primary care.	NA	Weitzman CC, Leventhal JM. Screening for behavioral health problems in primary care. *Curr Opin Pediatr*. 2006;18(6):641-648.
Interventions to improve children's access to mental health care: a	NA	Werlen L, Gjukaj D, Mohler-Kuo M, Puhan MA. Interventions to improve children's access to mental health care: a systematic review and meta-analysis. *Epidemiol Psychiatr Sci*. 2019;29:e58.

© 2023 Kass M et al. *JAMA Network Open*.

systematic review and meta-analysis.		
Adolescent depression screening in primary care: feasibility and acceptability.	Medical Staff	Zuckerbrot RA, Maxon L, Pagar D, Davies M, Fisher PW, Shaffer D. Adolescent depression screening in primary care: feasibility and acceptability. *Pediatrics*. 2007;119(1):101-108.
Perceptions and Attitudes Towards Pediatric Mental Health Screening		
Study Title	Sample Population	Full Citation
Online Behavioral Screener with Tailored Obesity Prevention Messages: Application to a Pediatric Clinical Setting.	Child-Parent Dyads	Chau S, Oldman S, Smith SR, Lin CA, Ali S, Duffy VB. Online Behavioral Screener with Tailored Obesity Prevention Messages: Application to a Pediatric Clinical Setting. *Nutrients*. 2021;13(1). doi:10.3390/nu13010223
Adapting Safety Check as a Universal Suicide Prevention Strategy in Pediatric Primary Care.	Parents	Davis M, Johnson C, Pettit AR, et al. Adapting Safety Check as a Universal Suicide Prevention Strategy in Pediatric Primary Care. *Acad Pediatr*. 2021;21(7):1161-1170.
Perception of primary care pediatricians of effectiveness, acceptability, and availability of mental health services.	Medical Staff	Dempster NR, Wildman BG, Duby J. Perception of primary care pediatricians of effectiveness, acceptability, and availability of mental health services. *J Child Health Care*. 2015;19(2):195-205.
Behavioral Health Needs, Barriers, and Parent Preferences in Rural Pediatric Primary Care.	Parents	Fehr KK, Leraas BC, Littles MMD. Behavioral Health Needs, Barriers, and Parent Preferences in Rural Pediatric Primary Care. *J Pediatr Psychol*. 2020;45(8):910-920.
Acceptability of Adolescent Social and Behavioral Health Screening	Patients (14- to 21-years)<br>Parents	Langerman SD, Badolato GM, Rucker A, Jarvis L, Patel SJ, Goyal MK. Acceptability of Adolescent Social and Behavioral Health Screening in the Emergency Department. *J Adolesc Health*. 2019;65(4):543-548.

© 2023 Kass M et al. *JAMA Network Open*.

in the Emergency Department.		
Patient and Caregiver Attitudes Towards Comprehensive Behavioral Health Screening in the Emergency Department.	Patients (14- to 21-years) Parents	Langerman S, Badolato GM, Rucker AC, Jarvis L, Patel SJ, Goyal MK. Patient and Caregiver Attitudes Towards Comprehensive Behavioral Health Screening in the Emergency Department. *Pediatrics*. 2018;142(1_MeetingAbstract):111-111.
Screening for Gender Identity in Adolescent Well Visits: Is It Feasible and Acceptable?	Patients (12-18-years)	Lau JS, Kline-Simon A, Sterling S, Hojilla JC, Hartman L. Screening for Gender Identity in Adolescent Well Visits: Is It Feasible and Acceptable? *J Adolesc Health*. 2021;68(6):1089-1095.
Development and initial validation of a measure of parents' preferences for behavioral counseling in primary care.	Parents	Riley AR, Walker BL, Hall TA. Development and initial validation of a measure of parents' preferences for behavioral counseling in primary care. *Fam Syst Health*. 2020;38(2):139-150.
Examining Caretaker Attitudes Towards Primary Prevention of Pediatric Behavioral Health Problems in Integrated Care.	Parents	Zimmermann M, O'Donohue W, Zepeda M, Woodley A. Examining Caretaker Attitudes Towards Primary Prevention of Pediatric Behavioral Health Problems in Integrated Care. *J Behav Health Serv Res*. 2021;48(1):120-132.
Validation of Mental Health Screening Tools		
Study Title	Sample Population	Full Citation
Comparison of the PSC-17 and alternative mental health screens in an at-risk primary care sample.	Parents	Gardner W, Lucas A, Kolko DJ, Campo JV. Comparison of the PSC-17 and alternative mental health screens in an at-risk primary care sample. *J Am Acad Child Adolesc Psychiatry*. 2007;46(5):611-618.
Screening for autism spectrum disorders in	Parents	Robins DL. Screening for autism spectrum disorders in primary care settings. *Autism*. 2008;12(5):537-556.

primary care settings.		

© 2023 Kass M et al. *JAMA Network Open*.

eFigure 1. Parents' Willingness to Discuss Mental Health and Learning Disorders.

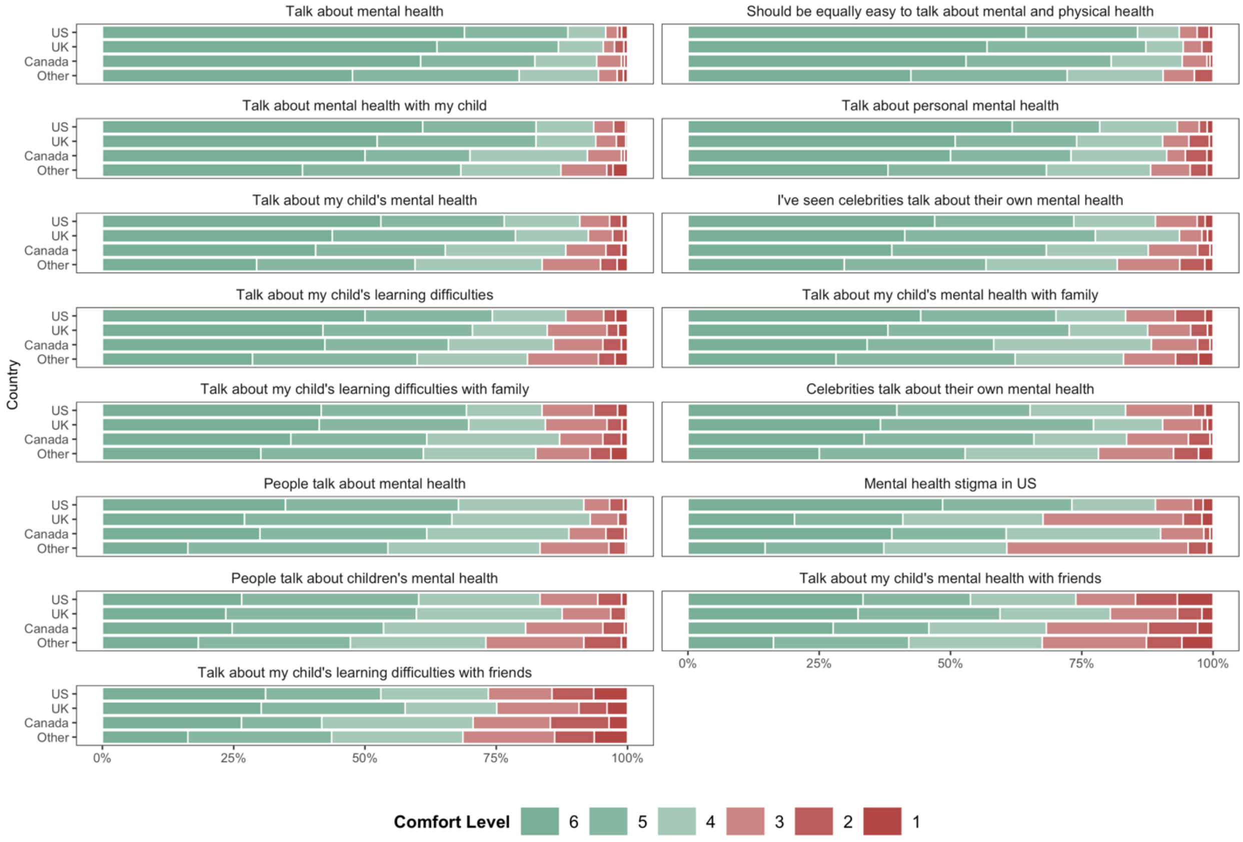

Parents' comfort levels on various statements about their willingness and/or ability to discuss mental health and learning disorders. The rating scale was a 6-point Likert scale, with 1=Disagree and 6=Agree.

© 2023 Kass M et al. *JAMA Network Open*.

eFigure 2. Mean Parental Comfort Levels of Screening Topics by Country.

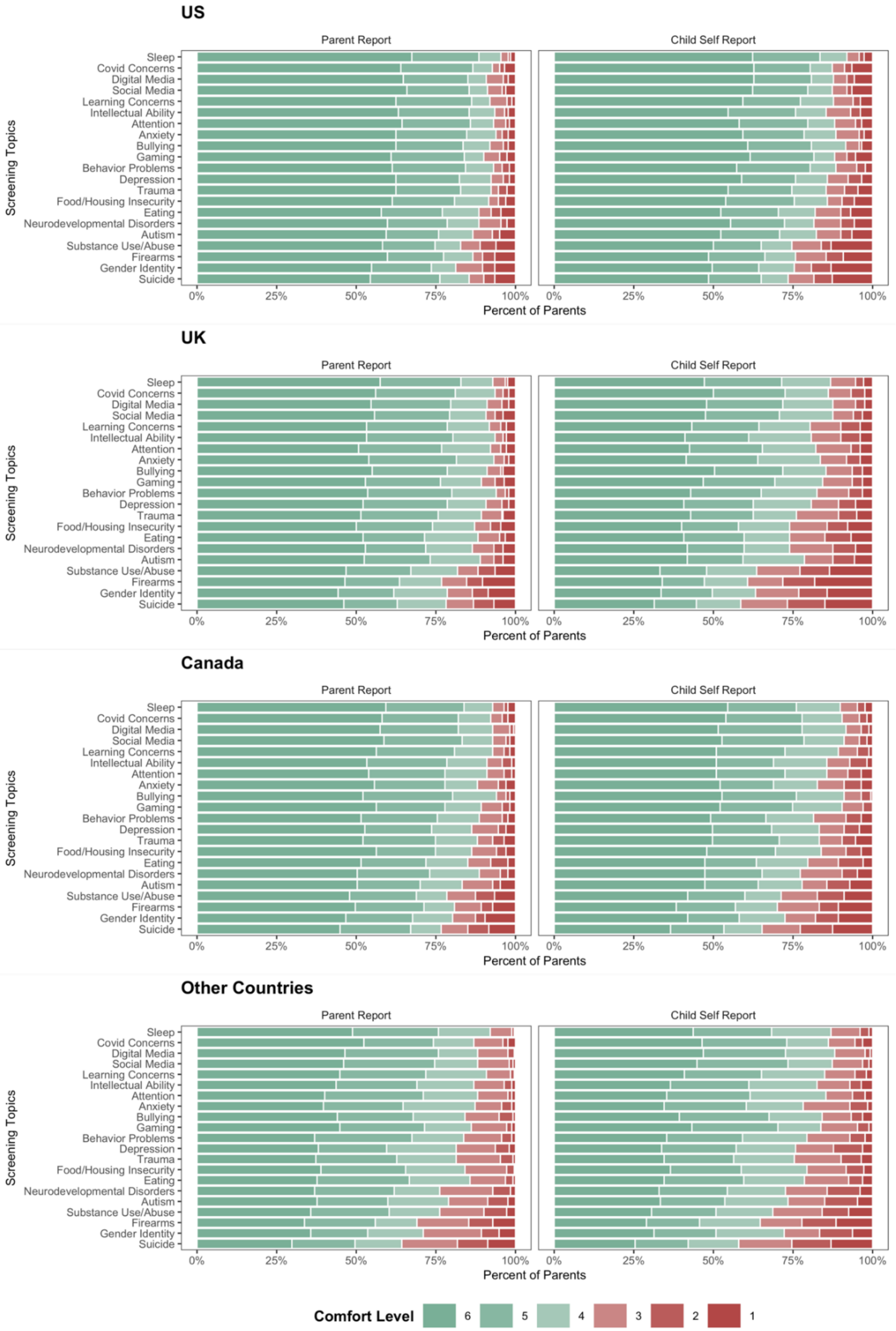

* *p*<.05, ***p*<.01, ****p*<.001.

© 2023 Kass M et al. *JAMA Network Open*.

Parental comfort levels of parent report and child self-report for various screening topics by country sample (US: *n*=265; UK: *n*=282; Canada: *n*=171; Other Countries: *n*=254). Topics are ordered according to the entire sample (*N*=972). Comfort levels ranged from 1 (not comfortable) to 6 (comfortable).

© 2023 Kass M et al. *JAMA Network Open*.

eFigure 3. Correlation of Child's Age and Parents' Comfort of Screening Topics by Report Option.

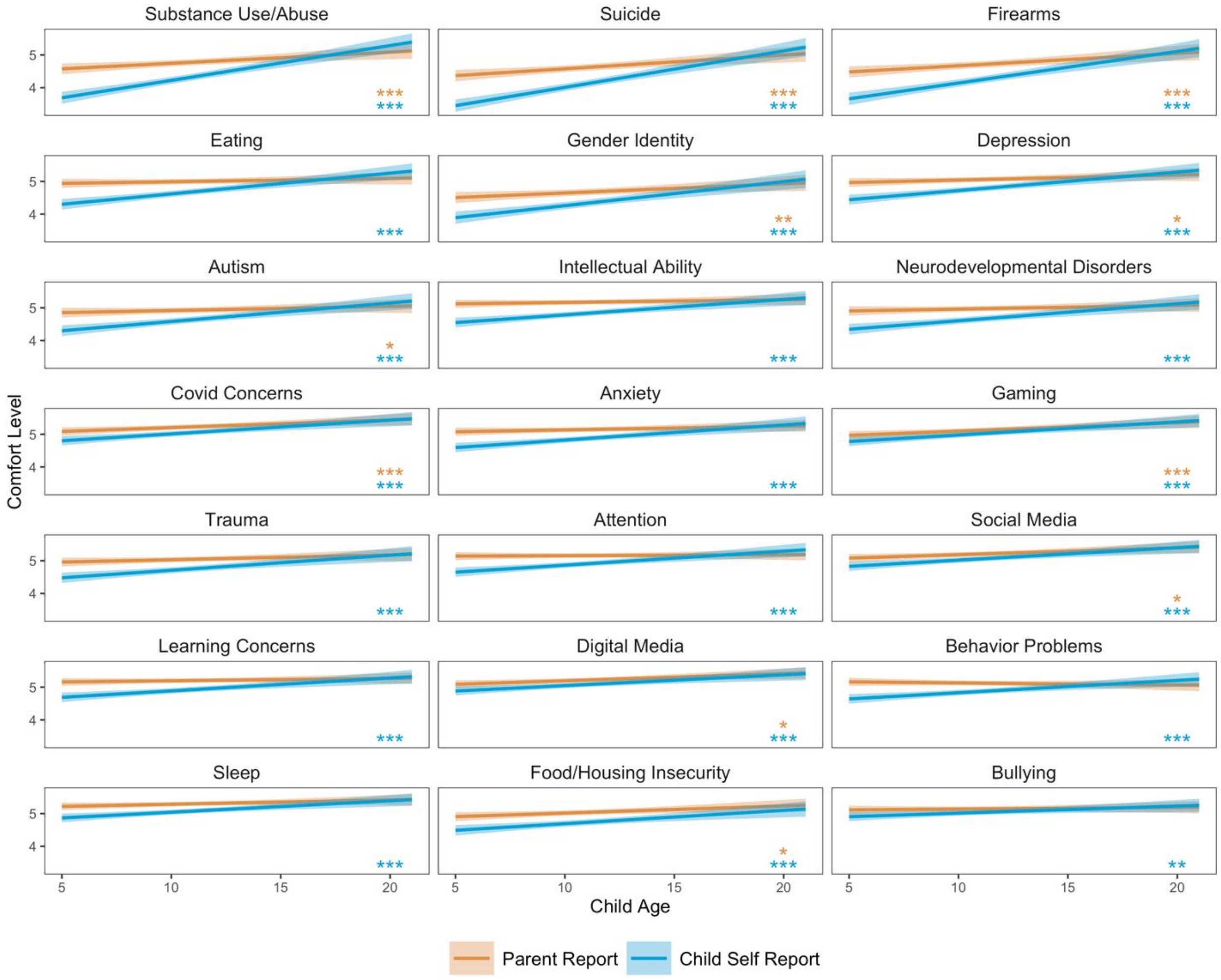

**p<.05, **p<.01, ***p<.001*

Results from a series of correlations of child age on parental comfort levels with each report option (parent- and child self-report) for all 21 topics. Topics are ordered by correlation coefficient of child self-report. Statistical significance is denoted by asterisks.

© 2023 Kass M et al. *JAMA Network Open*.
