# Supplementary material for: Parental Preferences for Mental Health Screening of Youths From a Multinational Survey
Source: JAMA Netw Open. 2023 Jun 20;6(6):e2318892. doi: 10.1001/jamanetworkopen.2023.18892 (PMC10282888; doi:10.1001/jamanetworkopen.2023.18892)
Supplement: Supplement 2. — Data Sharing Statement [file jamanetwopen-e2318892-s002.pdf]

Data Sharing Statement

Kass. Parental Preferences for Mental Health Screening of Youths From a Multinational Survey. *JAMA Netw Open*. Published June 20, 2023.
doi:10.1001/jamanetworkopen.2023.18892

Data
Data available: Yes
Data types: Deidentified participant data
How to access data: CMIDataUsage@childmind.org
When available: With publication

Supporting Documents
Document types: None

Additional Information
Who can access the data: anyone requesting the data
Types of analyses: for research purposes
Mechanisms of data availability: After approval of request form with brief proposal of research and data analysis plan
Any additional restrictions: None
